# Oral Mucosal Epithelial Cells Grown on Porous Silicon Membrane for Transfer to the Rat Eye

**DOI:** 10.1038/s41598-017-10793-1

**Published:** 2017-08-30

**Authors:** Yazad D. Irani, Sonja Klebe, Steven J. P. McInnes, Marek Jasieniak, Nicolas H. Voelcker, Keryn A. Williams

**Affiliations:** 1 Departments of Ophthalmology, Flinders University, Bedford Park, SA Australia; 2 Anatomical Pathology, Flinders University, Bedford Park, SA Australia; 3 Future Industries Institute, University of South Australia, Mawson Lakes, SA Australia; 4 Drug Delivery, Disposition and Dynamics, Monash Institute of Pharmaceutical Sciences, Monash University, Parkville, Victoria Australia; 5 Commonwealth Scientific and Industrial Research Organisation (CSIRO), Clayton, VIC Australia

## Abstract

Dysfunction of limbal stem cells or their niche can result in painful, potentially sight-threatening ocular surface disease. We examined the utility of surface-modified porous-silicon (pSi) membranes as a scaffold for the transfer of oral mucosal cells to the eye. Male-origin rat oral mucosal epithelial cells were grown on pSi coated with collagen-IV and vitronectin, and characterised by immunocytochemistry. Scaffolds bearing cells were implanted into normal female rats, close to the limbus, for 8 weeks. Histology, immunohistochemistry and a multiplex nested PCR for *sry* were performed to detect transplanted cells. Oral mucosal epithelial cells expanded on pSi scaffolds expressed the corneal epithelial cell marker CK3/12. A large percentage of cells were p63^+^, indicative of proliferative potential, and a small proportion expressed ABCG2^+^, a putative stem cell marker. Cell-bearing scaffolds transferred to the eyes of live rats, were well tolerated, as assessed by endpoint histology. Immunohistochemistry for pan-cytokeratins demonstrated that transplanted epithelial cells were retained on the pSi membranes at 8 weeks post-implant, but were not detectable on the central cornea using PCR for *sry*. The pSi scaffolds supported and retained transplanted rat oral mucosal epithelial cells *in vitro* and *in vivo* and recapitulate some aspects of an artificial stem cell niche.

## Introduction

The mammalian cornea is covered by specialised, non-keratinised epithelial cells that regenerate throughout life from adult stem cells located primarily at the limbus. Limbal stem cell deficiency, reflecting the loss or dysfunction of these adult stem cells, or disruption of the niche in which they reside, can result in painful and potentially blinding disease characterised by conjunctivalisation of the ocular surface, corneal vascularisation, and edema^1–7^.

Unilateral limbal stem cell deficiency can be treated by limbal transplantation using autologous tissue from the unaffected eye^8^. Bilateral disease necessitates allogeneic limbal stem cell transplantation, in which either tissue or *ex vivo*-expanded cells sourced from cadaveric human donors is used^9, 10^. Allogeneic limbal grafts tend to undergo immunological rejection, even under concurrent immunosuppression^11, 12^. Oral mucosa (OM) is an attractive alternative source of autologous cells for corneal surface regeneration as it is non-keratinised^13^, harbours adult epithelial stem cells^14, 15^, and can be relatively easily harvested^16^.

Proof-of-principle reports that autologous oral mucosal epithelial cells could restore the ocular surface in experimental animal models^17–19^ were followed in 2004 by a landmark report describing successful transfer of cell sheets, expanded from autologous buccal mucosa on amniotic membrane, to repair the damaged ocular surface in four human patients^20^. Oral mucosal epithelial cells obtained from biopsies and expanded on various scaffolds have since been transplanted as “bandages” to treat human ocular surface disease^21, 22^. However, the use of autologous oral mucosal epithelial cells to repopulate a stem cell niche has thus far received little attention. Reconstruction of such a niche requires not only a source of cells, but also an implantable scaffold to support the transplanted cells.

Porous silicon (pSi)^23^ exhibits some potential as a scaffold in regenerative medicine^24–28^. Psi membranes are inorganic, allowing efficient sterilization^29^, are biocompatible *in vitro* ^25, 30–32^ and *in vivo* ^30, 33^, degrade to non-toxic silicic acid^34^, can be surface-modified to improve degradation^35–37^ and attachment of cells^38, 39^, and can be loaded with proteins and growth factors^40^. We have previously shown that human corneal epithelial cells can be grown on pSi membranes coated with rat tail collagen, and that aminosilanised pSi membranes implanted under the rat conjunctiva do not erode the surrounding tissue^41^.

Herein, we describe the characterisation of rat oral mucosal epithelial cells grown on pSi membranes coated with collagen-IV and vitronectin, and the subsequent transplantation of the scaffolds into eyes of rats, in an attempt to recapitulate a limbal stem cell niche.

## Results

### Characterisation of pSi Membranes by SEM and ToF-SIMS

**Figure 1:**
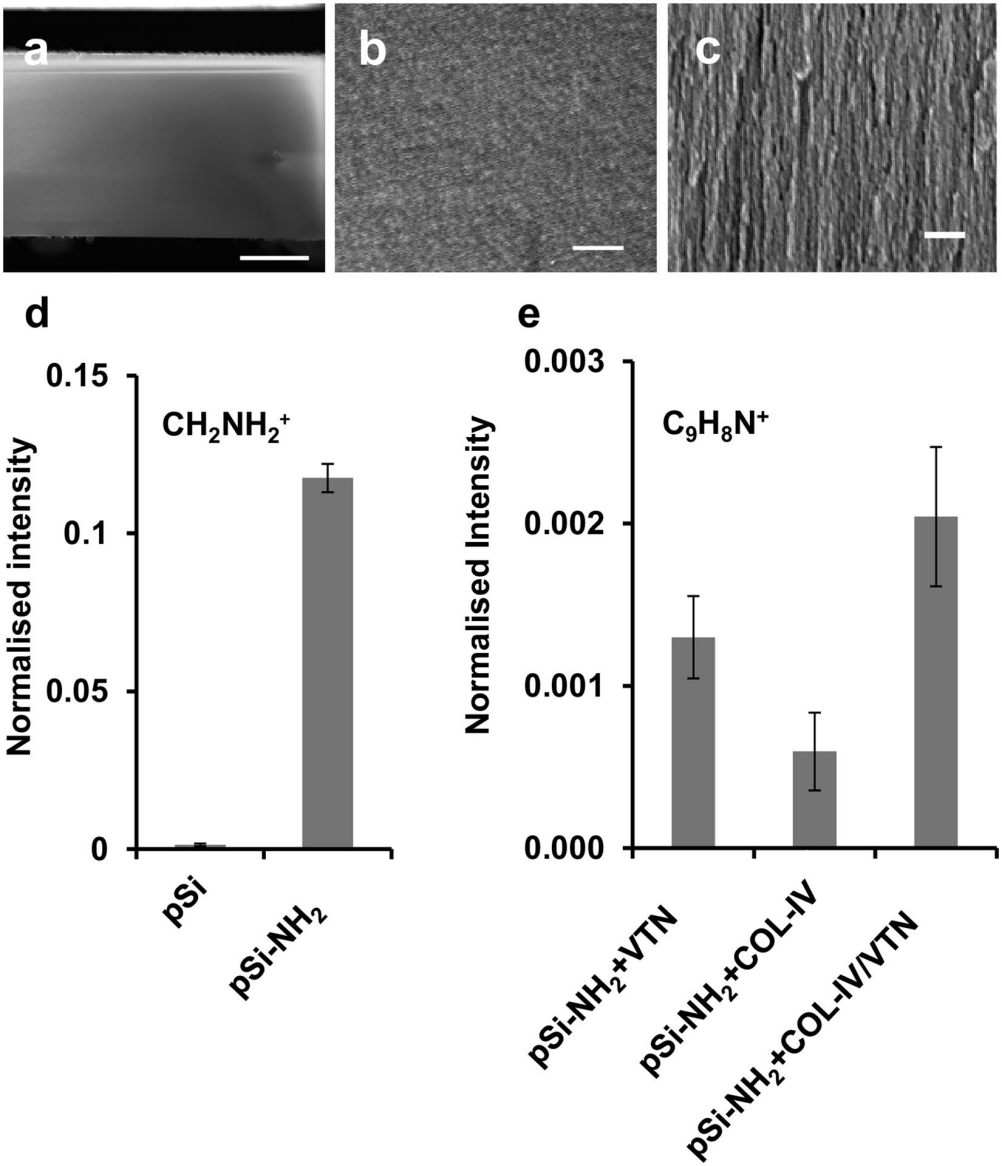
Characterization of pSi membrane materials. (**a**–**c**) Scanning electron micrographs of typical pSi membrane: (**a**) cross-sectional thickness, scale bar 100 µm; (**b**) surface pore structure *en face*, scale bar 1 µm; (**c**) pore structure extending through membrane, scale bar 1 µm. (**d**,**e**) Time-of-flight secondary ion mass spectrometry: (**d**) Normalised intensity of the C_2_H_2_N^+^ fragment ion (amine) on pSi membrane and aminosilanised pSi membrane (pSi-NH_2_) surfaces; (**e**) Normalised intensity of the C_9_H_8_N^+^ immonium ion on aminosilanised pSi membrane (pSi-NH_2_) coated with collagen-IV (COL), vitronectin (VTN), and collagen-IV plus vitronectin (COL-IV/VTN), indicative of surface loading.

Scanning electron microscopy (SEM) of the oxidised pSi membranes revealed a thickness of 276 µm (Fig. 1a) and an average pore size of 21.2 ± 6.7 nm (Fig. 1b). The pores extended from the surface through the entire thickness of the membrane (Fig. 1c). To confirm successful functionalisation with aminosilane (APTES), normalised intensities of the CH_2_NH_2_^+^ fragment ion on pSi membrane and its aminosilanised derivative pSi-NH_2_ were assessed by mass spectrometry (ToF-SIMS) (Fig. 1d). A narrow 95% confidence interval for the pSi-NH_2_ specimen indicated a homogeneous distribution of amine functional groups on the surface. To confirm surface adsorption of collagen-IV and vitronectin to pSi-NH_2_, ToF-SIMS was used to detect the C_9_H_8_N^+^ immonium ion on coated surfaces (Fig. 1e). Principal component analysis (PCA) applied to positive mass spectra allowed discrimination between collagen-IV and vitronectin adsorption (Supplementary Fig. S1).

### Characterisation of Rat Oral Mucosal Epithelial Cells

Epithelial cells harvested from the rat oral cavity were first plated on collagen-IV-coated glass coverslips, to establish their proliferative capacity. Over 7 days, the cells proliferated to form colonies with the cobblestone appearance characteristic of epithelial cells. Centrally, colonies contained densely packed cuboidal cells, whereas cells on the colony borders were more flattened. To examine the ability of pSi membranes to support the growth of oral mucosal epithelial cells, freshly isolated cells were stained with the cell tracker dye PKH26, plated on collagen-IV-coated pSi membranes and examined at 24 h, 48 h and 7 days. Cells survived, proliferated and formed colonies on the pSi scaffold (Supplementary Fig. S2).

**Figure 2:**
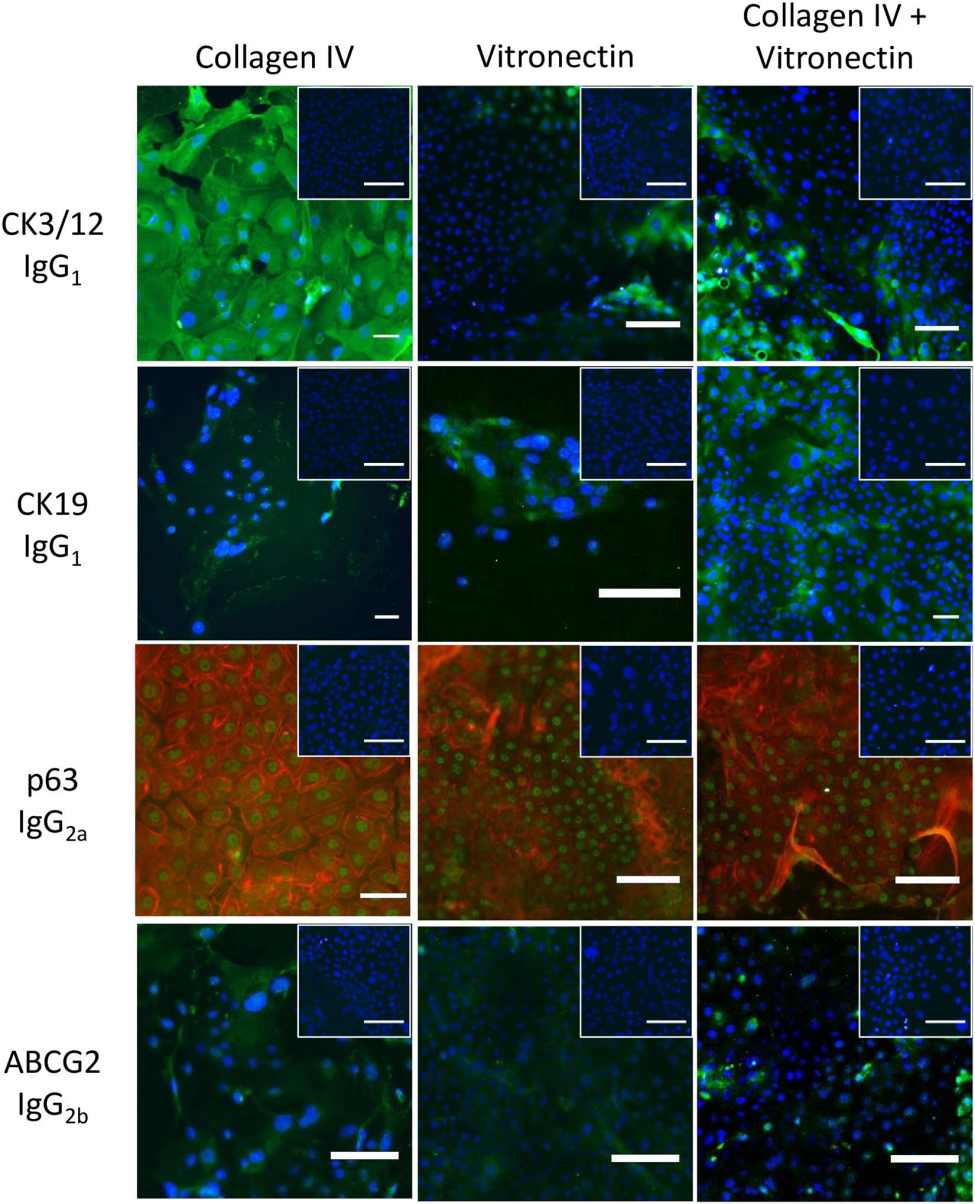
Phenotype of rat oral mucosal epithelial cells *in vitro*. Immunocytochemistry for CK3/12, CK19, p63 and ABCG2 on cells grown on pSi membranes coated with collagen IV (pSi-NH_2_ + COL-IV), vitronectin (pSi-NH_2_ + VTN), or collagen IV plus vitronectin (pSi-NH_2_ + COL-IV/VTN) at 7 days after seeding. Inserts, isotype-matched negative control antibodies (X63 for CK3/12 and CK19; Sal5 for p63 and ABCG2). Nuclei stained with Hoechst 33342 dye (blue), actin filaments stained with Alexa Fluor 594 phalloidin (red). Scale bars 50 µm.

To investigate the influence of different pSi membrane surface coatings on cell growth and phenotype, oral mucosal epithelial cells were plated on scaffolds coated with collagen-IV alone (pSi-NH_2_ + COL-IV), vitronectin alone (pSi-NH_2_ + VTN), or collagen-IV plus vitronectin (pSi-NH_2_ + COL-IV/VTN) and cultured for 7 days. Immunocytochemistry for corneal epithelial and putative stem cell markers was then performed (Fig. 2 and Table 1). Expression of CK3/12, a corneal epithelial differentiation marker, was variable but was most strongly expressed by cells grown on NH_2_ + COL-IV. Weak expression of CK19, a basal epithelial cell marker, was observed on cells grown on all coatings. Strong nuclear expression of the nuclear transcription factor p63, which labels transient amplifying cells, was observed in cells grown on all coatings. A small proportion of cells expressed the putative stem cell marker ABCG2. Because more cells expressed ABCG2 on pSi-NH_2_ + COL-IV/VTN than on those coated with either extracellular matrix protein alone (Table 1), all further experiments were performed with pSi-NH_2_ + COL-IV/VTN membranes.

**Table 1:** Summary of cytokeratin (CK) profile and putative stem cell marker expression in rat oral mucosal epithelial cells grown for 7 days on coated pSi membranes *in vitro*.

**pSi membrane coating**	**Expression in cultured oral mucosal epithelial cells of: ce cells**			
	**CK 3/12**	**CK 19**	**p63**	**ABCG2**
Collagen-IV	−/ + + +	+ weak	+ + +	−/+
Vitronectin	−/++	+ weak	+ + +	−/+
Collagen-IV + vitronectin	−/ + +	+ weak	+ + +	−/++

− No expression observed; + expressed in a few cells; + + expressed in a minority of cells; + ++ expressed in a majority of cells.

### Stability and Biocompatibility of Coated pSi Membranes *in vivo*

**Figure 3:**
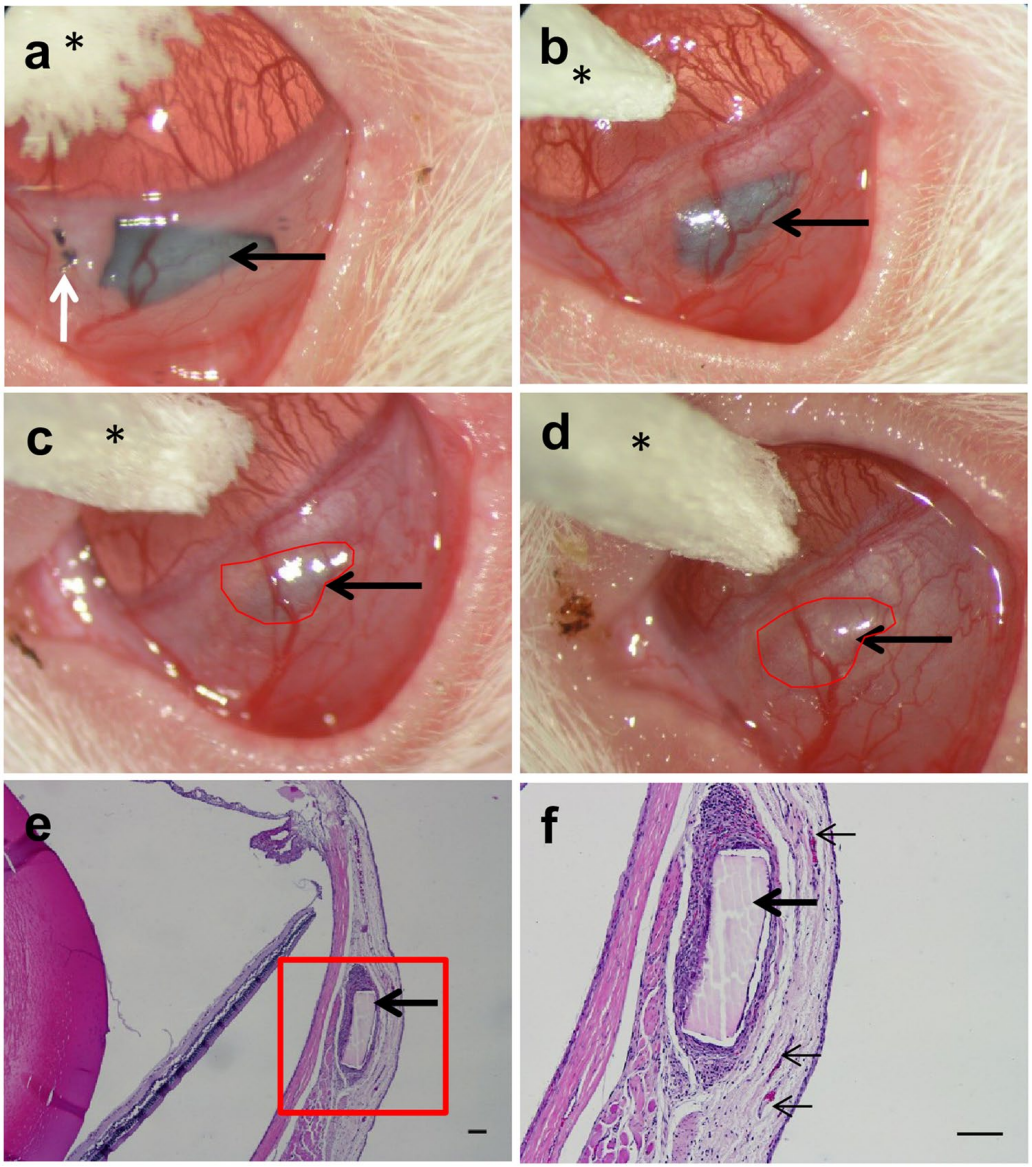
Collagen-IV plus vitronectin coated pSi membranes (pSi-NH_2_ + COL-IV/VTN) were implanted under the conjunctiva of Sprague-Dawley rats. (**a**) pSi membrane (black arrow) visible at one day after implantation (white arrow marks the sutures). (**b**) Two weeks post implantation, mild inflammation and neovascularisation were apparent. (**c**) Four weeks post implantation, signs of degradation were apparent (scaffold colour changed from black to brown). (**d**) Eight weeks after implantation, the pSi membrane was still visible. An ophthalmic spear (asterisk) is visible at the top of images (**a**–**d)**. The outline of the pSi membrane is traced with a red line in images (**c**,**d**). Images captured at the operating microscope. (**e**) Endpoint histology. Haematoxylin and eosin stained section of a rat eye showing the pSi membrane at 8 weeks post-implantation. Inflammatory cells are visible around the implant. Scale bar 100 µm. (**f**) Higher magnification of the area depicted by the box in image E. Some blood vessels (small black arrows) are seen in the vicinity of the implant. Scale bar 100 µm.

pSi-NH_2_ + COL-IV/VTN membranes (without cells) were implanted into the subconjunctival space near the limbus of two female rats and followed for 8 weeks. The pSi membrane was clearly visible post-implantation (Fig. 3a). The implants caused mild erythema for up to two weeks, and new vessels grew in the vicinity of the implant (Fig. 3b). By four weeks post-implant, no residual inflammation was apparent. The pSi membrane underwent some degradation, as evidenced by the change in colour from black to brown (Fig. 3c). However, the implant remained visible at the operating microscope up to 8 weeks post-implantation (Fig. 3d). The new vessels around the site of the implant underwent remodelling with some regression (Fig. 3d). Endpoint histology confirmed the pSi membranes were still present at 8 weeks post-implant (Fig. 3e). Some inflammatory cells, indicative of a foreign body giant cell response, were observed at the implant site. There was no evidence of a fibrous capsule surrounding the membrane, although some blood vessels were observed in the vicinity (Fig. 3f).

### Transplantation of Oral Mucosal Cells on pSi Membranes to The Rat Eye

**Figure 4:**
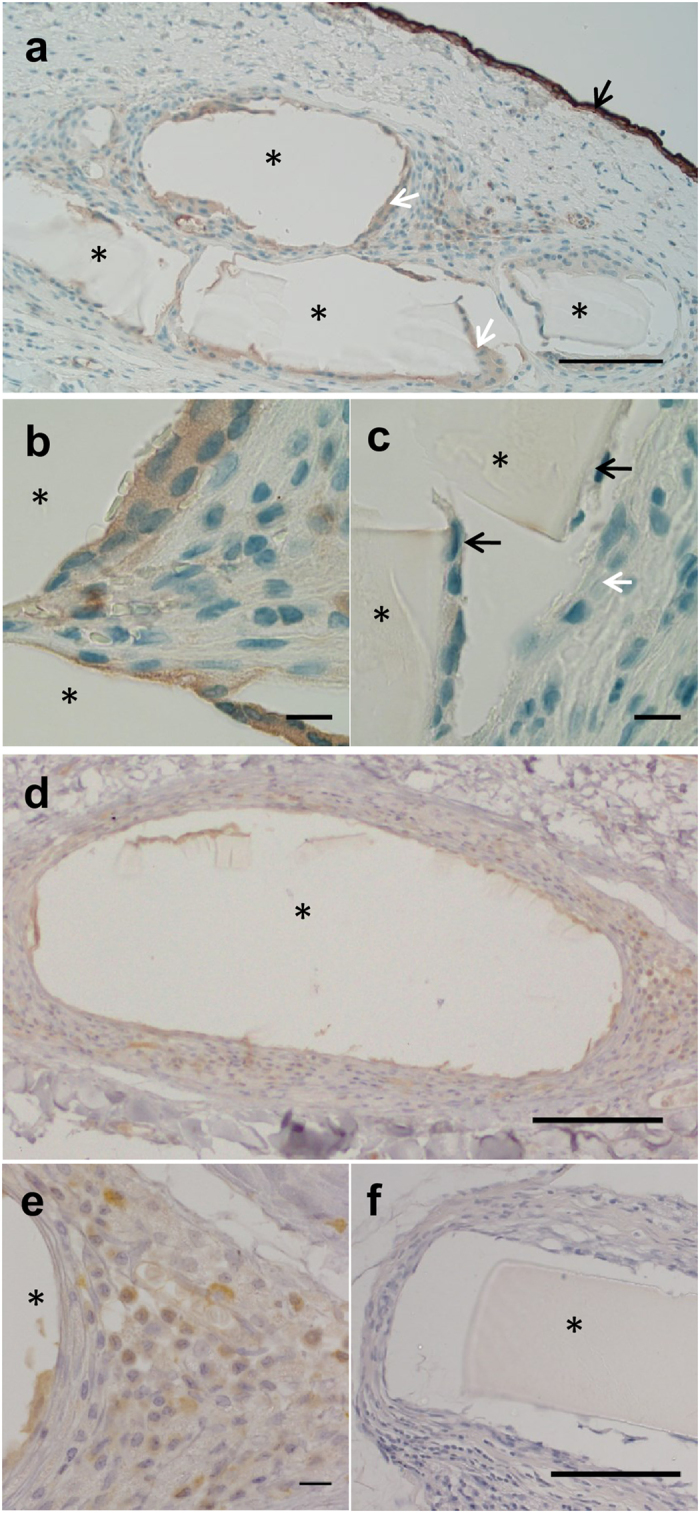
Immunohistochemistry for cytokeratins to detect transplanted epithelial cells on coated pSi membranes at 8 weeks post-implant. (**a**) Cells weakly positive for pan-cytokeratin (white arrows) on the surface of the implant (asterisk). Strong expression of pan-cytokeratin is normally present in conjunctival epithelium (black arrow), serving as an internal positive control. (**b**) Higher magnification showing labelled cells on the surface of the implant. (**c**) pSi membranes *without* cells were implanted under the conjunctiva of rats for 8 weeks. The cells in contact with the pSi (black arrows) did not express pan-cytokeratin, indicating they were not epithelial cells. (**d**) Cells in the vicinity of the pSi implant demonstrated positive labelling for cytokeratin 14. (**e**) Higher magnification showing CK14 labelled cells. (**f**) Tissue surrounding pSi membranes implanted *without* oral mucosal cells, was not positive for CK14. Scale bars for panels a and d 100 µ, and panels b, c, e, f 10 µm.

pSi-NH_2_ + COL-IV/VTN membranes carrying 7 day cultured oral mucosal cells were implanted at the limbus of rats and followed for 8 weeks. Endstage immunohistochemistry for cytokeratins was then performed, to detect transplanted epithelial cells remaining on the scaffolds. Weak expression of both pan-cytokeratin and cytokeratin 14, a basal epithelial marker, was observed in cells at the edge of the pSi implant (Fig. 4a,b,d,e), suggesting that transplanted cells had survived on the implant and migrated locally. No cytokeratin expression was observed on control coated pSi membranes implanted *without* oral mucosal epithelial cells (Fig. 4c,f). The cells surrounding the implant did not express the transient amplifying cell marker p63 (Supplementary Fig. S3). We then performed labelling for the histiocyte marker CD163 to determine if the cells surrounding the implant consisted of epitheliod inflammatory cells. Sparse labelling for CD163 was observed (Supplementary Fig. S4) indicating the majority of cells were not histiocytes. Isotype matched antibodies were used as negative controls (Supplementary Fig. S5).

**Figure 5:**
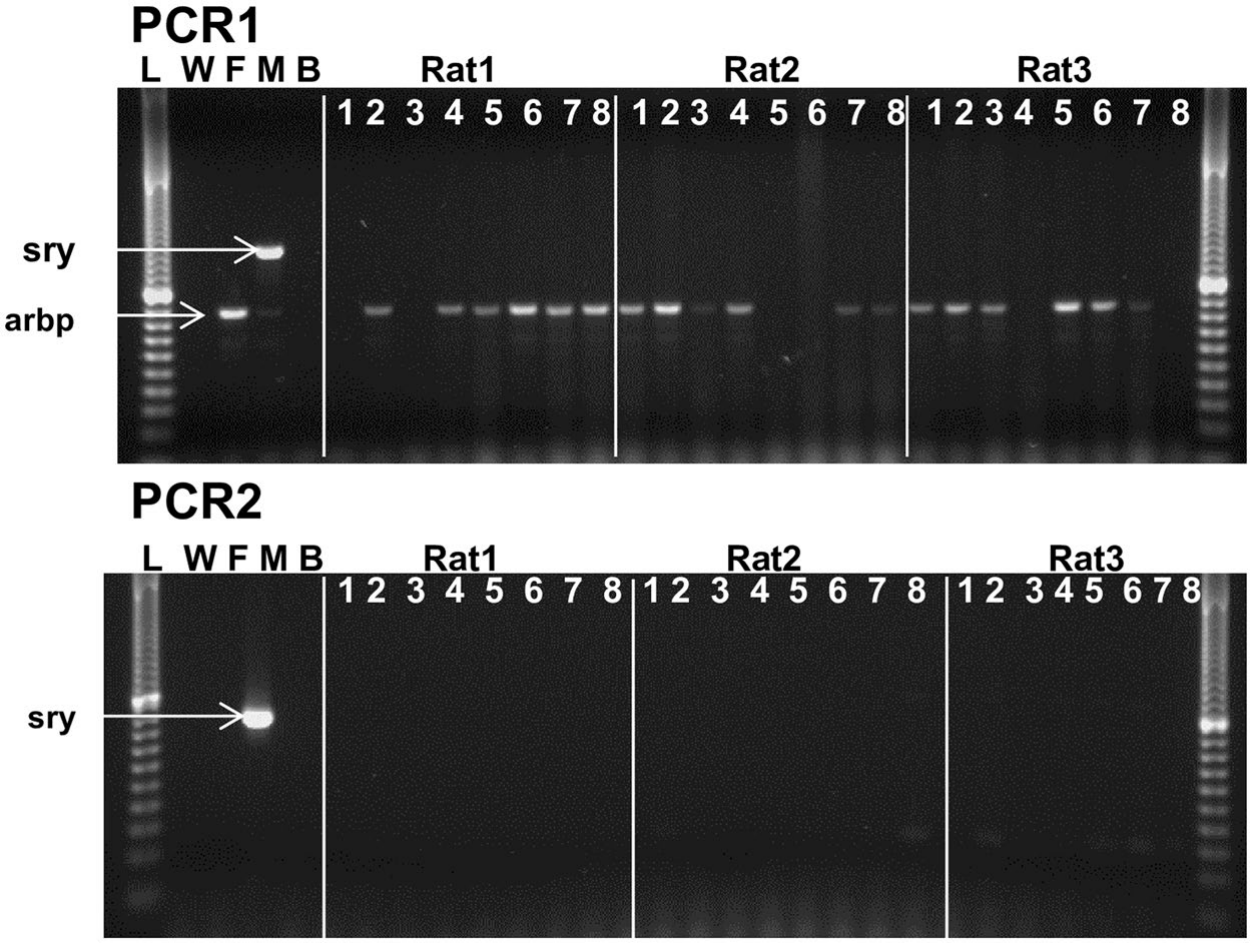
Failure to detect transplanted male oral mucosal epithelial cells on the central cornea of female rats. Male oral mucosal cells grown on coated pSi membrane scaffolds were implanted at the limbus of female rats. Cell samples were collected weekly from the cornea using FTA paper. The presence of transplanted male cells was detected by PCR for the male-specific *sry* gene. No male cells were detected on the surface of the corneas of the 3 female rats. L = 20 base pair ladder, W = water control, F = female rat genomic DNA, M = male rat genomic DNA, B = blank FTA disc, 1–8 = sample taken x weeks after transplantation.

Migration of the transplanted cells across the corneal surface was assessed by impression cytology from the central cornea using FTA paper, followed by amplification of the male specific marker *sry* by PCR. As male cells were transplanted into female rats, amplification of the male specific gene *sry* allowed detection of the transplanted cells. The sensitivity of this assay allowed detection of a single male cell in the presence of female cells. A band corresponding to the expected size for *arbp*, a housekeeper gene, was detected in 17/24 (70%) samples tested, indicating that the FTA paper retrieved cells from the corneal surface and preserved amplifiable DNA from the majority of samples. That the male specific gene sry failed to amplify in any test sample (Fig. 5) indicated that no implanted donor cells were present on the central corneal surface. These data suggested that the rat oral mucosal epithelial cells supported on pSi scaffolds and transplanted to the limbus had not migrated to the central cornea.

## Discussion

The transfer of a population of epithelial progenitor cells to the surface of the cornea as a living bandage, whether or not supported on a scaffold such as amniotic membrane, will not necessarily lead to long-term repair of a damaged ocular surface if the limbal stem cell niche has been irreparably damaged. Regeneration of such a niche requires first, a biocompatible scaffold that can be implanted surgically into the eye so that the integrity of the surrounding tissue is not compromised; second, incorporation of factors within the artificial niche that can retain and support the “stemness” of at least a proportion of the cells seeded within it, and third, a source of cells with capacity for self-renewal, proliferation and differentiation, to populate the artificial niche.

We considered that small pieces of pSi membrane might have potential for the construction of an artificial niche because of their ease of fabrication from inorganic silicon, biocompatibility within the eye, capacity for surface modification, and ability to support mammalian cell attachment and growth^40–43^. The porous structure mimics to some extent the retes of the palisades of Vogt, and lends the material a large surface area into which bioactive factors can be loaded. The opacity of a pSi membrane yields some protection from incident light and is not a disadvantage, given that the material is to be implanted at the limbus, well away from the visual axis. A disadvantage might be the relative stiffness of the material.

Oral mucosa has already proved to be a valuable source of autologous epithelial stem cells for ocular bandages in human patients, for purposes of ocular surface repair and relief of pain^14, 44, 45^, and was the source of stem cells in this study. For development of a suitable experimental model in which to test the performance of an artificial limbal stem cell niche, we chose the rat. The availability of inbred strains, such as the inbred Sprague-Dawley used in this study, allowed transfer of cells from donors to recipients as histocompatible isografts. Because immune rejection will not occur, the need for concomitant immunosuppression can be avoided. Rat oral mucosal cells have been isolated previously by others, and shown to be fit for purpose^46^. Furthermore, rat oral mucosal cells exhibit reasonable proliferative potential^47^.

In our hands, rat oral mucosal cells cultured *ex vivo* on pSi membranes consisted of a heterogeneous population of cells in varying degrees of differentiation. CK3/12, which is expressed by human mature central corneal epithelial cells^48^ but not found in basal limbal epithelial cells^49^, was expressed strongly in some cells while others exhibited low or no expression. Rat oral mucosal cells expressed low levels of CK19, a marker present in basal epithelial cells of the human peripheral cornea but not in the central cornea^50^. p63 is expressed in human keratinocyte stem cells^51^. In human corneas, the transcription factor p63 localises to basal epithelial cells with higher expression at the limbus^52^, and is thought to label transient amplifying cells. A majority of rat oral mucosal cells exhibited strong nuclear expression of p63, indicating that the cells had most likely maintained their proliferative potential when cultured on pSi scaffolds. The most widely-used marker for putative corneal epithelial stem cells is the cell membrane-bound transporter protein, ABCG2^53–58^. A small proportion of the cells expanded on pSi scaffolds expressed ABCG2, indicative of the presence of a small number of putative stem cells. In summary, the population of cultured rat oral mucosal cells contained some differentiated cells that expressed CK3/12, a large number of p63-positive transient amplifying cells, and a small number of stem cells that expressed ABCG2. We considered this mixture of cells to be appropriate for seeding an artificial stem cell niche.

The detailed proteome of the human limbal stem cell niche is still to be defined, however recent evidence suggests that corneal epithelial stem cells carry the vitronectin receptor, αvβ5 integrin^54^, and that corneal, limbal and conjunctival basement membranes all contain collagen type IV is well-established^55^. Vitronectin and collagen-IV were thus used separately and in conjunction to coat pSi membranes designed to support oral mucosal cell attachment and growth. Because more cells expressed ABCG2 on pSi-NH_2_ + COL-IV/VTN membranes than on those coated with either extracellular matrix protein alone, we selected the combination for further experimentation.

In deciding where to implant the artificial niche, we considered available literature. In the mouse, corneal oligopotent cells are widely distributed throughout the corneal epithelium^56^, but more recent data suggest that functionally, the limbus is the major repository for corneal epithelial stem cells^57^. Rather less is known about the corneal epithelial stem cell niche in the rat, but the available evidence would point to locations within the limbus or palpebral conjunctiva^58, 59^. We thus chose to implant pSi-NH_2_ + COL-IV/VTN membranes carrying cultured rat oral mucosal cells under the bulbar conjunctiva, as close as possible to the corneolimbal junction. Male donor cells were transferred to the eyes of female recipient rats to enable tracking of any migration of transplanted cells carrying *sry*: we have previously reported no influence of gender on limbal isograft survival in the rat^11^.

Although some oral mucosal cells remained on the implanted pSi membranes after 8 weeks, as evidenced by labelling for cytokeratins, we were unable to detect transplanted cells in the central cornea by *sry* PCR^11, 60^. Our assay was able to detect 8 pg of male genomic DNA, equivalent to 1 cell, in the presence of female cells. Under normal conditions, corneal epithelial cells migrate from the periphery to the centre of the cornea in 7 to 10 days^61, 62^. The length of the current study was 8 weeks, which might have provided sufficient time for the cells to migrate to the central cornea, especially as detection of transplanted cells on the scaffold at 8 weeks post-implant indicated their continuing presence. However, our corneas were normal, not wounded. Our findings point to the partial reconstruction of a niche capable of maintaining epithelial cells.

## Materials and Methods

### pSi Membranes

pSi was prepared as described previously^63^, except that current density of 166.7 mAcm^−2^ was applied for 60 min. Membranes of pSi were separated from bulk silicon by electropolishing for 4 min in 1:20 hydrofluoric acid: ethanol at 4 mAcm^−2^, oxidised in a tube furnace (LABEC, Marrickville, NSW, Australia) for 1 hour at 600 °C, and aminosilanised with APTES as described previously^41^.

### Scanning Electron Microscopy (SEM)

SEM was performed with an ultra-high-resolution field emission gun MERLIN microscope with GEMINI II column (Carl Zeiss Microscopy GmbH, Jena, Germany) operating in high-resolution mode at accelerating voltage 2 kV. Secondary electron imaging was performed with a parallel on-axis in-lens secondary electron detector. The working distance was maintained at 2 mm.

### Time-of-flight Secondary Ion Mass Spectrometry (ToF-SIMS)

ToF-SIMS measurements were performed with a PHI TRIFT V nanoTOF instrument (PHI Electronics Ltd, Chanhassen, MN, USA). A 30 keV, pulsed primary^197^Au^+^ ion beam was used to sputter and ionise species from each sample surface. Positive mass axis calibration was performed with CH_3_^+^, C_2_H_5_^+^ and C_3_H_7_^+^ whilst CH^−^, C_2_H^−^ and F^−^ were used to calibrate negative mass axis. Spectra were acquired under static SIMS conditions^64^. A mass resolution m/Δm of >7000 at nominal m/z = 27 amu (C_2_H_3_^+^) was typically achieved. Each sample was characterized by five positive ion mass spectra, collected from non-overlapping areas. Multiple mass spectra were processed by principal component analysis (PCA) and Analysis of Means^65^.

### Coating of Porous Silicon Membranes

PSi membranes were coated with 10 µg human collagen-IV (Sigma-Aldrich, St. Louis, MO, USA), or 1 µg rat vitronectin (Sigma-Aldrich), or both collagen-IV and vitronectin, as previously described^42^.

### Experimental Animals

Inbred male and female Sprague-Dawley rats sourced from the Flinders University Animal Facility were exposed to a 12 hour light-dark cycle and allowed access to food and water *ad libitum*. All animal experiments were approved by the Animal Welfare Committee of Flinders University, and were in accordance with the ARVO Statement for the Use of Animals in Ophthalmic and Vision Research and the Australian Code of Practice for the Care and Use of Animals for Scientific Purposes.

### Harvest and Culture of Rat Oral Mucosal Epithelial Cells

Oral mucosal epithelial tissue was harvested from 4–6 week old male rats. Tissue was washed with balanced salt-antibiotic solution (BSS, Alcon, Fort Worth, TX, USA; supplemented with 200 IU/ml penicillin, 200 µg/ml streptomycin and 10 ng/ml amphotericin; Life Technologies, Carlsbad, CA, USA) and placed in one ml MCDB153 medium (Sigma-Aldrich) with 2.2 U/ml dispase I (Life Technologies) for 1 h at 37 °C. The epithelial tissue was gently detached from underlying tissue, washed in sterile BSS and placed in 500 µl 0.25% trypsin, 2.65 mM EDTA (Sigma-Aldrich) in BSS for 5 min. The tissue was gently disaggregated in one ml MCDB153 with 10% vol/vol fetal bovine serum (FBS), insulin transferrin sodium selenate supplement (Life Technologies), 100 U/ml penicillin/100 µg/ml streptomycin, 5 ng/ml amphotericin, 5 ng/ml epidermal growth factor (EGF) (Prospec Tany, Rehovot, Israel), 10 ng/ml β-nerve growth factor (NGF) (R&D Systems, Minneapolis, MN, USA), 200 ng/ml hydrocortisone (Sigma-Aldrich) and 0.03 pg/ml tri-iodothyronine (Sigma-Aldrich) (“complete medium”). Cells were washed with 10 ml complete medium and pelleted at 100 *g* for 5 min, resuspended in complete medium, seeded on protein-coated aminosilanised pSi membranes in 24 well plates, and incubated at 37 °C with 5% CO_2_ in air. Cells were used at passage one. One-two donor rats were used per experiment. Cells were stained using the cell tracker dye PKH26 (Sigma-Aldrich) as described previously^41^.

### Surgical Implantation of pSi Scaffolds into the Rat Eye

Protein-coated pSi membranes (1–2 mm^2^), with or without cells attached, were implanted under the bulbar conjunctiva, at the limbus, of female Sprague-Dawley rats as described elsewhere^41, 43^.

### Immunocytochemistry

Immunocytochemistry was performed on rat oral mucosal epithelial cells grown on pSi scaffolds as previously described^41^. Murine monoclonal antibodies with specificity for CK3/12 (clone AE5, IgG1, Millipore, Billerica, MA, USA), CK19 (clone E6,IgG1, Abcam, Cambridge, United Kingdom), p63 (clone 4A4, IgG2a, Abcam), and ABCG2 (clone 5D3, IgG2b, Chemicon, Temecula, CA, USA) were used at a dilution of 1/100 in phosphate-buffered saline with 1% vol/vol normal goat serum (Sigma-Aldrich). Negative controls were isotype-matched antibodies (X63: IgG1; Sal5: IgG2a) of irrelevant specificity. The secondary antibody was a goat anti-mouse IgG conjugated to Alexa Fluor 488 (Life Technologies). Cell nuclei were counterstained with 2 µg/ml Hoechst 33342 (Sigma-Aldrich) for 5 min at room temperature. Where the protein of interest localised to the nucleus, cytoplasmic actin was stained with 1/200 Alexa Fluor 594 phalloidin (Life Technologies).

### Endpoint Histology

Whole eyes were removed from rats 8 weeks post-pSi implantation, fixed in 10% buffered formalin, embedded in paraffin wax, sectioned and stained with haematoxylin and eosin^40^. All sections were assessed by a qualified pathologist (SK).

### Immunohistochemistry

Formalin-fixed, paraffin-embedded tissue was sectioned at 6 µm thickness on a microtome (Leica, Wetzlar, Germany). Pan-cytokeratin antibody cocktail (Clones AE1/AE3) was sourced from Cell Marque (Rocklin, CA, USA). Cytokeratin 14 antibody was obtained from Abcam (clone RCK107, IgG_1_) and the CD163 antibody was purchased from Leica (clone 10D6, IgG_1_). Primary antibodies were diluted according to the manufacturer’s instructions. The Novolink polymer detection system (Leica, Wetzlar, Germany) was used to detect positive labelling according to the manufacturer’s instructions.

### Impression Cytology and sry PCR

A 2 mm disc of FTA paper was applied to the central cornea, and gently agitated in a circular motion to collect superficial cells. The FTA discs were then placed in a sterile tube for later analysis by PCR. A two-step PCR protocol to amplify the male-specific *sry* gene was employed to detect transplanted cells. The first multiplexed step amplified the housekeeper gene *arbp* (to confirm presence of DNA in the samples) and the *sry* gene. The sensitivity of the detection system was enhanced using a second round of amplification using nested primers for *sry*. Each reaction consisted of 17.4 µl water, 2.5 µl 10X PCR buffer, 0.75 µl MgCl_2_, 0.25 µl dNTPs, 2 µl primer mix (20:1 ratio of ARBP and SRY1 primers, Table 2), and 0.1 µl Platinum Taq DNA polymerase (Life Technologies). Two µl of male rat genomic DNA was used as a positive control. FTA discs were added to the PCR mix and subjected to thirty cycles of denaturation at 95 °C, annealing at 62 °C and extension at 72 °C. The results of the PCR were visualised by separating 5 µl of the PCR product on a 2% agarose gel. Two microliters of the first PCR product (diluted 1/100) was used as the template in the second reaction. The PCR mix was prepared identically except that the primers SRY2 and SRY2rev were used (Table 2). An annealing temperature of 60 °C was used for the second reaction.

**Table 2:** Primers used to detect the male-specific gene *sry*.

Primer	Sequence (5′ → 3′)	Description
SRY1for	TGCATTTATGGTGTGGTCCCG	sry multiplex primer
SRY1rev	CTGTGTAGGGTCTTCAGTCTCTGC	sry multiplex primer
SRY2for	TGTCTAGATAGCATGGAGGGC	Second round primer
SRY2rev	CCTCTGTGGCACTTTAACCCTT	Second round primer
ARBPfor	CCATCTGCATTTGCGGC	Housekeeping gene
ARBPrev	GCAGGCTGACTTGGTGTGA	Housekeeping gene

## Electronic supplementary material

Supplementary Figures
