# Supplementary material for: Oral Mucosal Epithelial Cells Grown on Porous Silicon Membrane for Transfer to the Rat Eye
Source: Sci Rep. 2017 Aug 30;7:10042. doi: 10.1038/s41598-017-10793-1 (PMC5577150; doi:10.1038/s41598-017-10793-1)
Supplement: Supplementary file 1 — Supplementary Figures [file 41598_2017_10793_MOESM1_ESM.pdf]

Oral Mucosal Epithelial Cells Grown on Porous Silicon Membrane for Transfer to the Rat Eye

Yazad D. Irani,[a,*] Sonja Klebe,[b] Steven J. P. McInnes,[c] Marek Jasieniak,[c] Nicolas H. Voelcker,[c,d,e] and Keryn A. Williams[a]

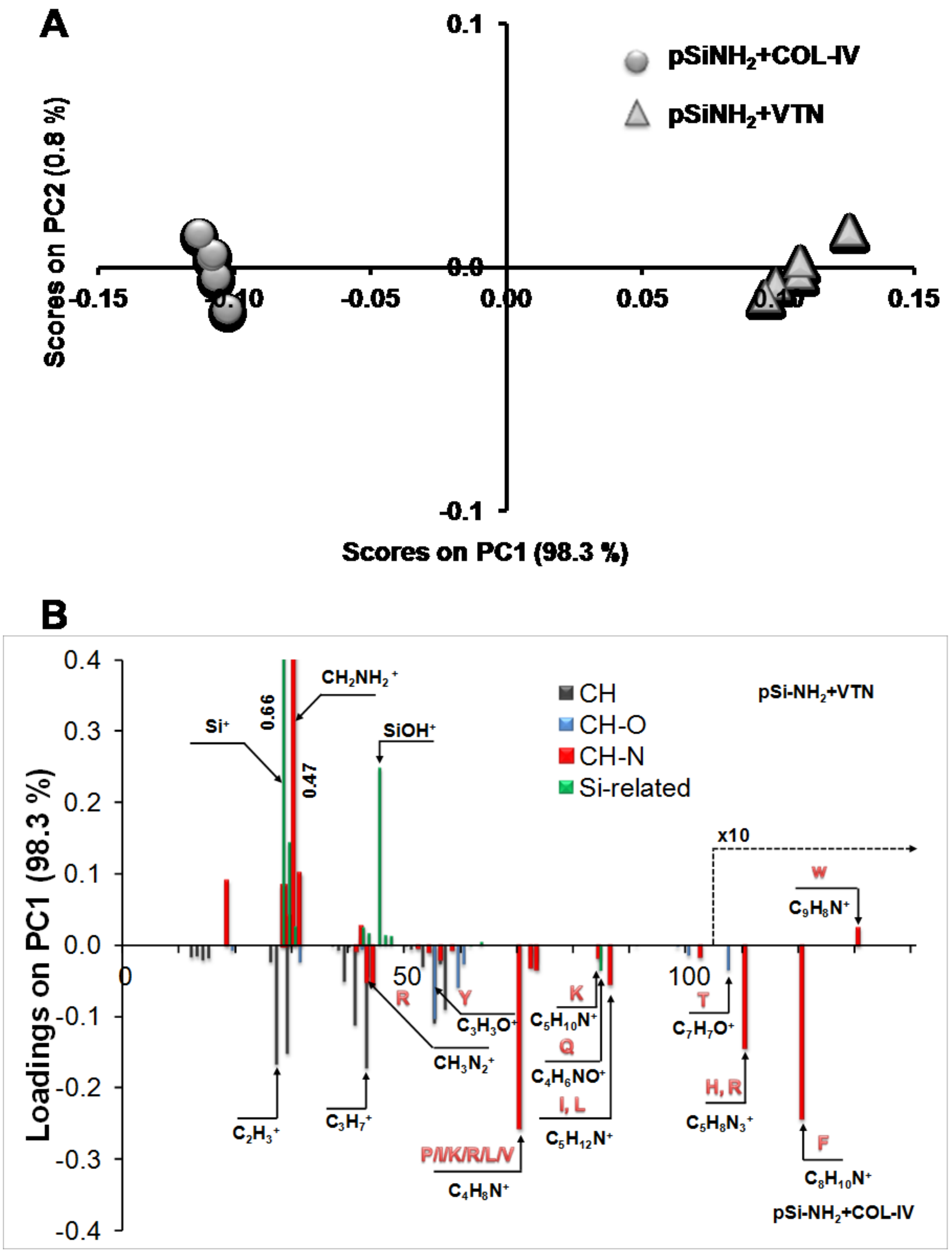

Supplementary Fig S1

Principal component analysis (PCA) across pSi-NH_2+collagen-IV (COL-IV) and pSi-NH_2+vitronectin (VTN) surfaces; (A) scores plot of positive mass spectra on PC1 and PC2; (B) loadings of positive fragment ions on PC1. PCA applied to positive mass

spectra yielded two matrices expressed by scores (trace a) and loadings (trace b) plots. The scores plot of the spectra on PC1 and PC2 showed two well-separated groups along PC1, capturing 98% of the original data variance. Surface chemistries of pSi-NH_2+COL-IV and pSi-NH_2+VTN were different but similar within each group. Loadings of positive fragment ions on PC1 revealed that immonium ions characteristic of F, H, I, K, L, P, R, Q, T, V amino acids loaded negatively on to PC1. The scores plot points indicated they were associated with COL-IV adsorbed on pSi-NH_2. The $C_9H_8N^+$ positive fragment ion, which is a fingerprint of tryptophan (W), was the only immonium ion that loaded positively on PC1 and was characteristic of the pSi-NH_2 surface coated with the VTN solution. Thus, both collagen-IV and vitronectin adsorbed to the aminosilanised pSi.

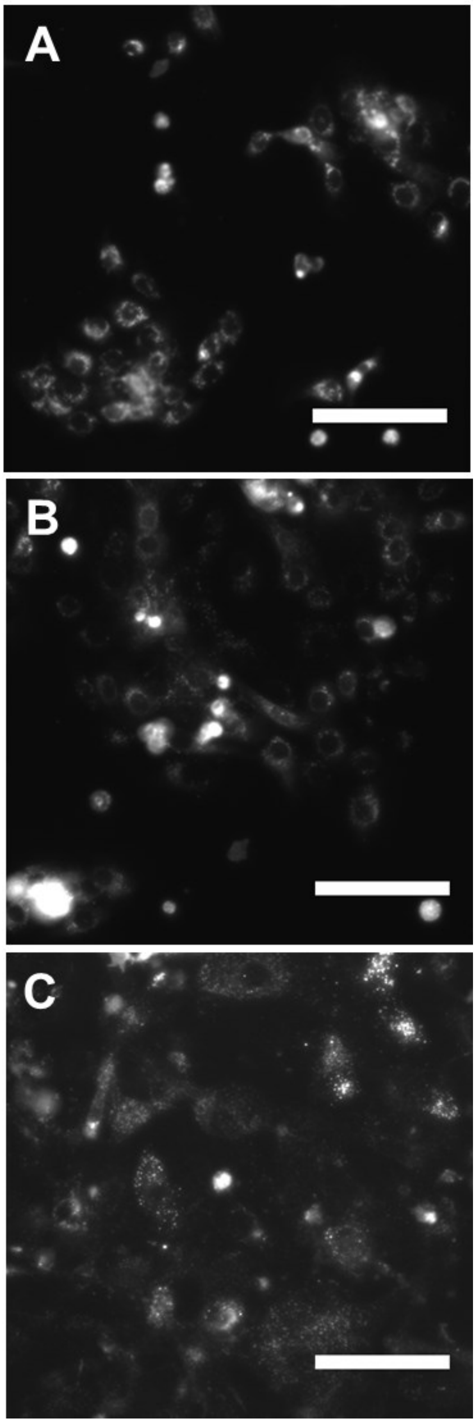

Supplementary Fig S2

PKH26 staining of oral mucosal epithelial cells grown on collagen-IV-coated pSi membranes. Cells formed colonies on pSi membranes at: (A) 24 hours after seeding; (B) 48 hours after seeding; and (C) 7 days after seeding. Scale bars 100 μm. White arrows show individual cells and the white circle demarcates a colony of cells.

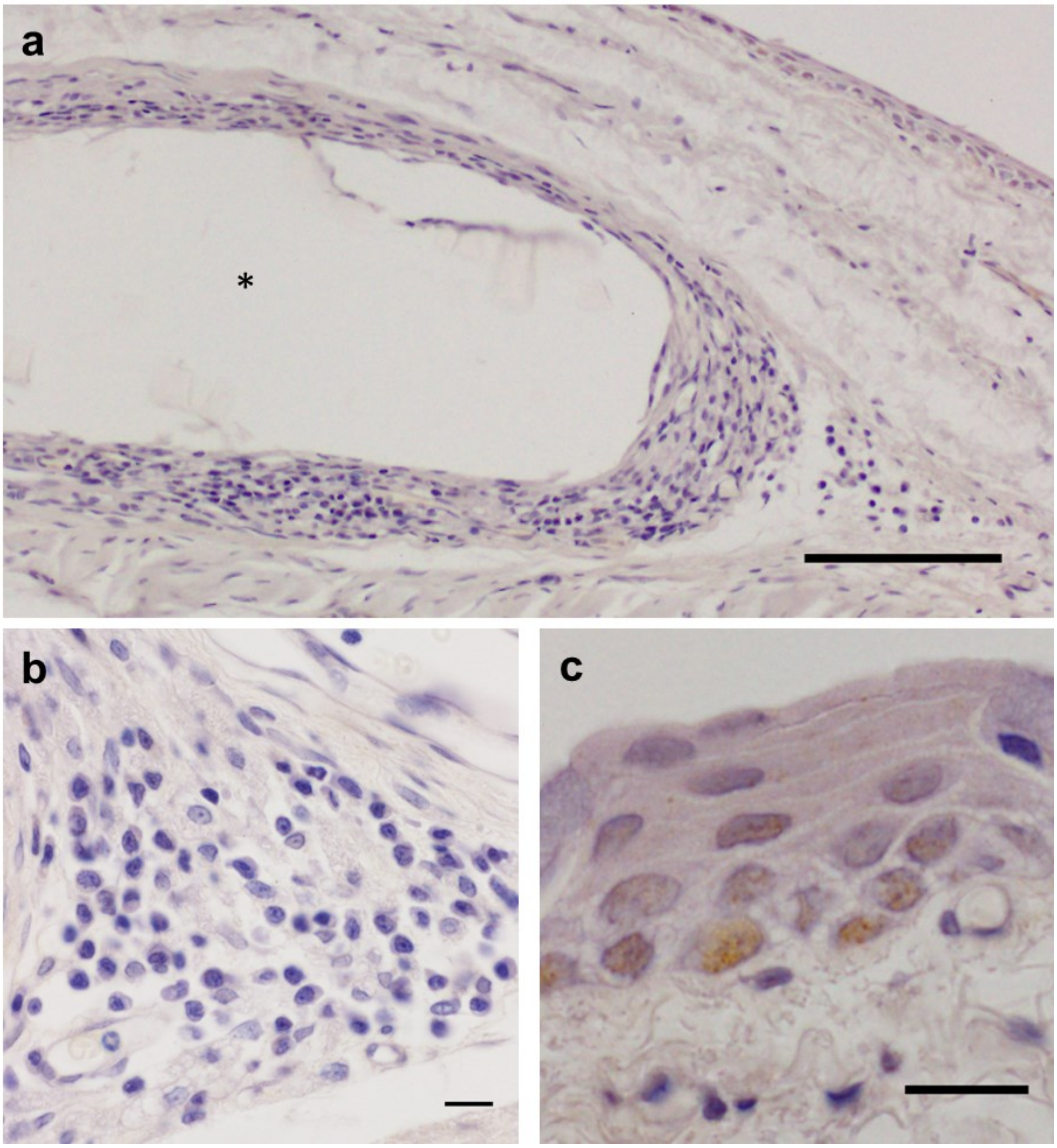

Supplementary Fig S3

Immunohistochemistry for the transient amplifying cell marker p63, 8 weeks post-transplant. (a) p63 was not detected in the cells surrounding the pSi implant (asterix). (b) high magnification (100X) image of cells surrounding pSi implant. (c) positive labelling for p63 in basal epithelial cells at the limbus. Scale bar (a) 100 µm, (b and c) 10 µm.

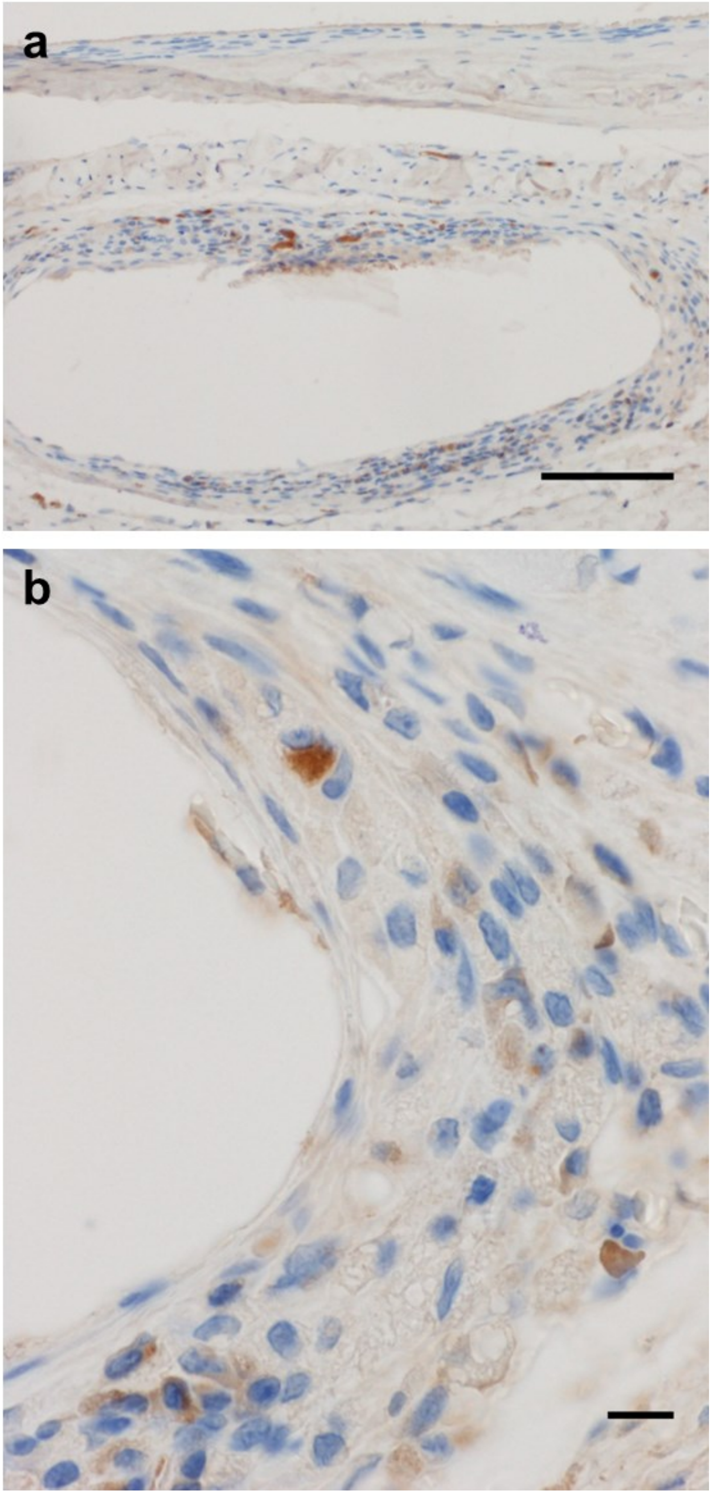

Supplementary Fig S4

Immunohistochemistry for the histiocytes marker CD163, 8 weeks post-transplant. (a) A small proportion of cells surrounding the implant labelled positive for CD163. (b) Higher magnification (100X) showing labelled cells. These data indicate that the majority of the cells surrounding the implant were not histiocytes.

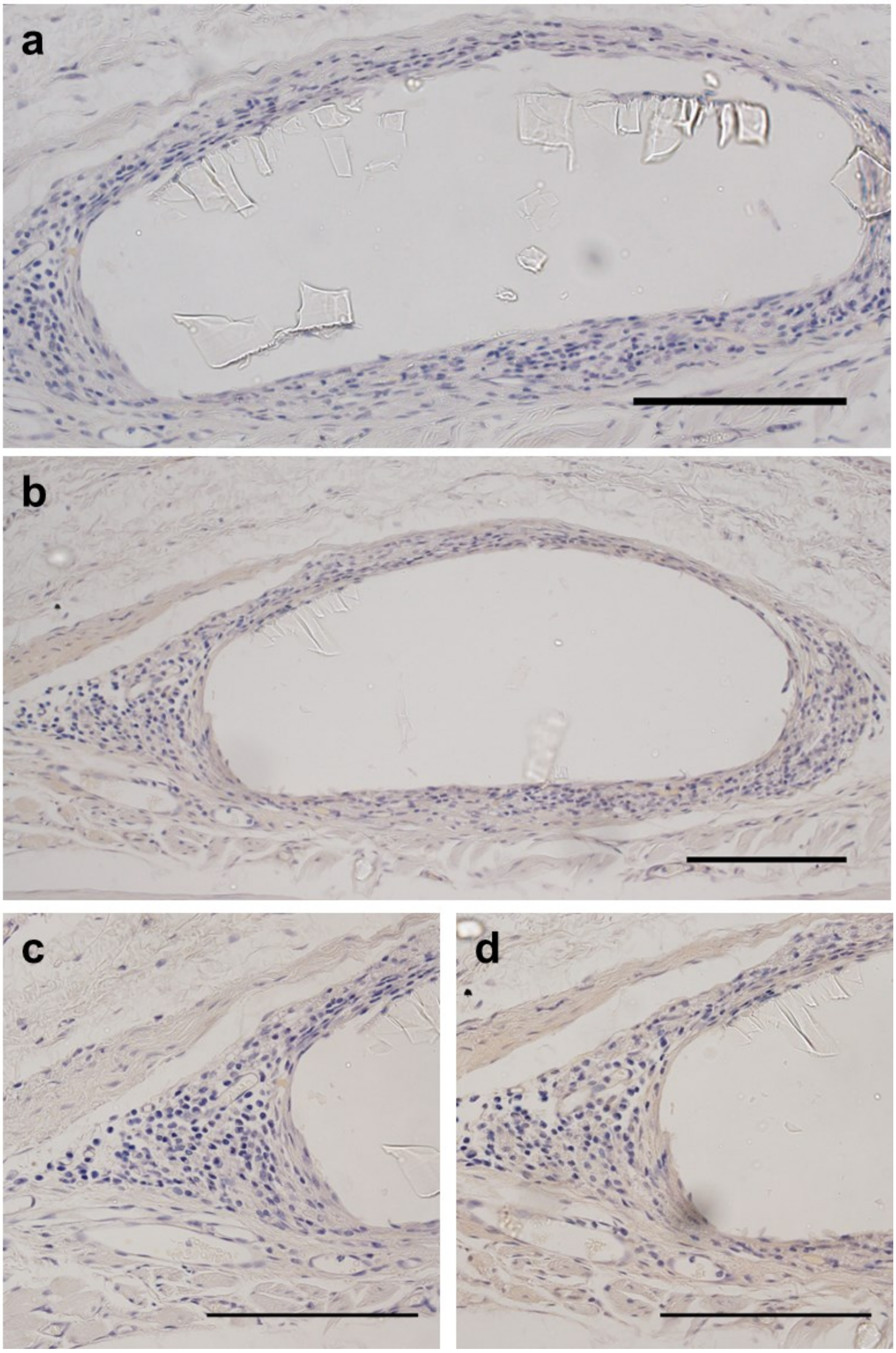

Supplementary Fig S5

Isotype-matched antibodies were used as negative controls for immunohistochemistry. Coated pSi membranes carrying oral mucosal cells were implanted under the conjunctiva of rats for 8 weeks. Formalin-fixed paraffin-embedded tissue was sectioned and labelled with isotype-matched antibodies X63 (IgG_1, panel a and c) and SAL5 ($IgG_{2a,}$ panel b and d). Scale bars 100 μm).
